# *Porphyromonas gingivalis* gingipains cause defective macrophage migration towards apoptotic cells and inhibit phagocytosis of primary apoptotic neutrophils

**DOI:** 10.1038/cddis.2016.481

**Published:** 2017-03-02

**Authors:** Sowmya A Castro, Russell Collighan, Peter A Lambert, Irundika HK Dias, Parbata Chauhan, Charlotte E Bland, Ivana Milic, Michael R Milward, Paul R Cooper, Andrew Devitt

**Affiliations:** 1 School of Life & Health Sciences and Aston Research Centre for Healthy Ageing, Aston University, Birmingham B4 7ET, UK; 2 School of Dentistry, University of Birmingham, Edgbaston, Birmingham B5 7EG, UK

## Abstract

Periodontal disease is a prevalent chronic inflammatory condition characterised by an aberrant host response to a pathogenic plaque biofilm resulting in local tissue damage and frustrated healing that can result in tooth loss. Cysteine proteases (gingipains) from the key periodontal pathogen *Porphyromonas gingivalis* have been implicated in periodontal disease pathogenesis by inhibiting inflammation resolution and are linked with systemic chronic inflammatory conditions such as rheumatoid arthritis. Efficient clearance of apoptotic cells is essential for the resolution of inflammation and tissue restoration. Here we sought to characterise the innate immune clearance of apoptotic cells and its modulation by gingipains. We examined the capacity of gingipain-treated macrophages to migrate towards and phagocytose apoptotic cells. Lysine gingipain treatment of macrophages impaired macrophage migration towards apoptotic neutrophils. Furthermore, lysine gingipain treatment reduced surface expression levels of CD14, a key macrophage receptor for apoptotic cells, which resulted in reduced macrophage interactions with apoptotic cells. Additionally, while apoptotic cells and their derived secretome were shown to inhibit TNF-*α*-induced expression by *P. gingivalis* lipopolysaccharide, we demonstrated that gingipain preparations induced a rapid inflammatory response in macrophages that was resistant to the anti-inflammatory effects of apoptotic cells or their secretome. Taken together, these data indicate that *P. gingivalis* may promote the chronic inflammation seen in periodontal disease patients by multiple mechanisms, including rapid, potent gingipain-mediated inflammation, coupled with receptor cleavage leading to defective clearance of apoptotic cells and reduced anti-inflammatory responses. Thus, gingipains represent a potential therapeutic target for intervention in the management of chronic periodontal disease.

Tightly regulated tissue homeostasis is an essential physiological process, which is maintained through a fine balance of cell proliferation, cell differentiation and cell death. The process of apoptosis that occurs during an inflammatory response supports the resolution of inflammation by the secretion of anti-inflammatory cytokines and pro-resolving molecules, which block further inflammatory cell infiltration and promotes recruitment of phagocytes which restore tissue homeostasis. Recent work demonstrates that a variety of factors promote the clearance of apoptotic cells (AC) and that these mediate different stages within a complex multistage process of apoptotic cell clearance by professional Apoptotic cell-derived extracellular vesicles and soluble factors (collectively known as the apoptotic cell secretome) can promote recruitment of phagocytes to sites of cell death whereupon ligand–receptor interactions enable tethering and engulfment of cell corpses.^3, 4, 5, 6^ Failure in any one of the stages of AC clearance can lead to inflammatory disease as a consequence of secondary necrosis of AC, due to release of intracellular antigens and immune stimulation.^7, 8, 9^

In the oral cavity, gingival tissues are exposed to a wide range of microorganisms. Evidence indicates that local tissue apoptosis drives the regulation of immune-inflammatory reactions, which occur in response to microbes producing anti-inflammatory signals affecting phagocytes at the site of infection.^10^ Defective control of the inflammatory response in this complex microenvironment can lead to the chronic, hyper-inflammatory disease of periodontitis. Notably, *Porphyromonas gingivalis* has been associated with inducing and propagating this aberrant host response.^11^ The non-resolving hyper-inflammatory response results in local tissue damage and ultimately tooth loss. The disease is also associated with several chronic inflammatory systemic diseases. *P. gingivalis* reportedly impairs the host inflammatory response, which underpins periodontal disease pathogenesis.^12, 13, 14^

A range of virulence factors are expressed by *P. gingivalis*, including the important cysteine-proteases gingipains, which may be arginine-specific (RgpA and RgpB) or lysine-specific (Kgp). Gingipains play a major role in the onset of inflammation by multiple mechanisms including: modifying the complement system; matrix metalloproteinase activity; neutrophil function; periodontal tissue vascular structure and responses; the host cytokine network and cell surface receptor levels.^15^ Notably, gingipains have been shown to cleave key pattern recognition receptors,^16, 17^ which have been shown to mediate recognition and removal of AC.^18^ Previous work has now also demonstrated that products of *P. gingivalis* may modulate AC clearance via multiple changes at the apoptotic cell surface.^19^ Here we address the hypothesis that gingipains promote disease progression by acting to inhibit multiple stages of the AC clearance process. We assess the effect of gingipains on the ability of macrophages to identify AC, interact with and remove AC and respond to resolve inflammation. This study therefore provides novel insights into potential mechanisms important in periodontitis progression.

## Results

### Purification and characterisation of proteolytic enzymes from *P. gingivalis*

**Figure 1:**
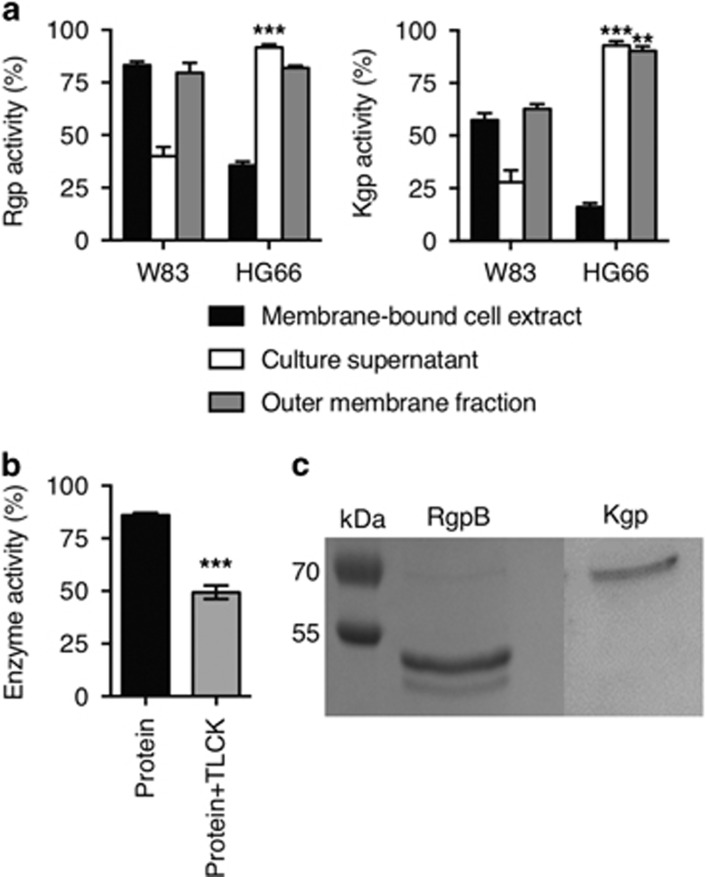
Determination of amidolytic activity and molecular weight of purified proteolytic enzymes from *P. gingivalis*. Strains W83 or HG66 were grown and cultures fractionated as described. Enzyme amidolytic activity was assessed (**a**) for Rgp activity against Bz-L-Arg-pNA (left panel) and, for Kgp activity, against Z-L-LYS-pNA (right panel) for: membrane-bound cell extract (black bar), cell-free culture supernatant (white bar) and outer membrane fractions (grey bar). Statistical tests compared differences between strains. Enzymes were purified using chromatography. (**b**) Compares the TLCK inhibition as a measure of cysteine protease activity in the purified gingipains. (**c**) SDS-PAGE electrophoresis of *P. gingivalis* HG66 purified Rgp and Kgp gingipain. Statistical analyses were conducted using ANOVA followed by Bonferroni *post hoc-*test *P<0.05, **P<0.01, ***P<0.001. Data shown are mean±S.E.M. of three independent experiments of log phase bacterial cultures grown for 24 h (O.D. 600 nm)

Preliminary experiments to isolate gingipains were undertaken using two strains of *P. gingivalis* (strains W83 and HG66). Cultures were fractionated and the presence of gingipain was assessed using an assay of protease activity in membrane, culture supernatant and outer membrane fractions (Figures 1a and b). These assays revealed that both gingipain forms (Rgp and Kgp) were released into culture supernatant at significantly higher active amounts by strain HG66 compared with strain W83 (Figure 1a). Subsequently, culture supernatants from strain HG66 were used as a source of both Rgp and Kgp and the enzymes were purified using gel filtration and affinity chromatography with Sephadex G-150 and arginine-Sepharose. Following purification, cysteine protease activity was confirmed using the specific inhibitor TLCK, which is specific for trypsin-like proteases such as Kgp and Rgp (Figure 1b). Molecular mass analysis (Figure 1c) of the purified proteins indicated a single major band for Kgp and a double band for Rgp of anticipated molecular mass of ≃60 kDa and ≃50 kDa, respectively.^20, 21^ Purification of gingipains was confirmed using mass spectrometric analysis (Supplementary Table 1). Lipopolysaccharide (LPS) contamination was assessed within the purified gingipain fractions using the Limulus Amoebocyte Lysate assay, which confirmed endotoxin presence at levels 2.9 U/ml (RgpB) and 1.9 U/ml (Kgp). Following successful purification of gingipains, the impact of these important pathogen-derived enzymes on the removal of AC was assessed.

### Gingipains reduce expression of CD14, an apoptotic cell clearance receptor

A range of phagocyte receptors have been implicated in the recognition of AC and CD14, an important innate immune component, has been identified as a key tethering receptor for AC.^22, 23^ Our previous studies have demonstrated that the human monocyte cell line THP-1, when differentiated, provides an effective model for studying the role of macrophages (MØ) in AC clearance and to assess the role of cytokines and other inflammatory mediators released during this process.^5, 24, 25^ To understand the impact of gingipains on the expression of important phagocyte receptors, we examined a panel of innate immune receptors on phagocytes before and after treatment with gingipains.

**Figure 2:**
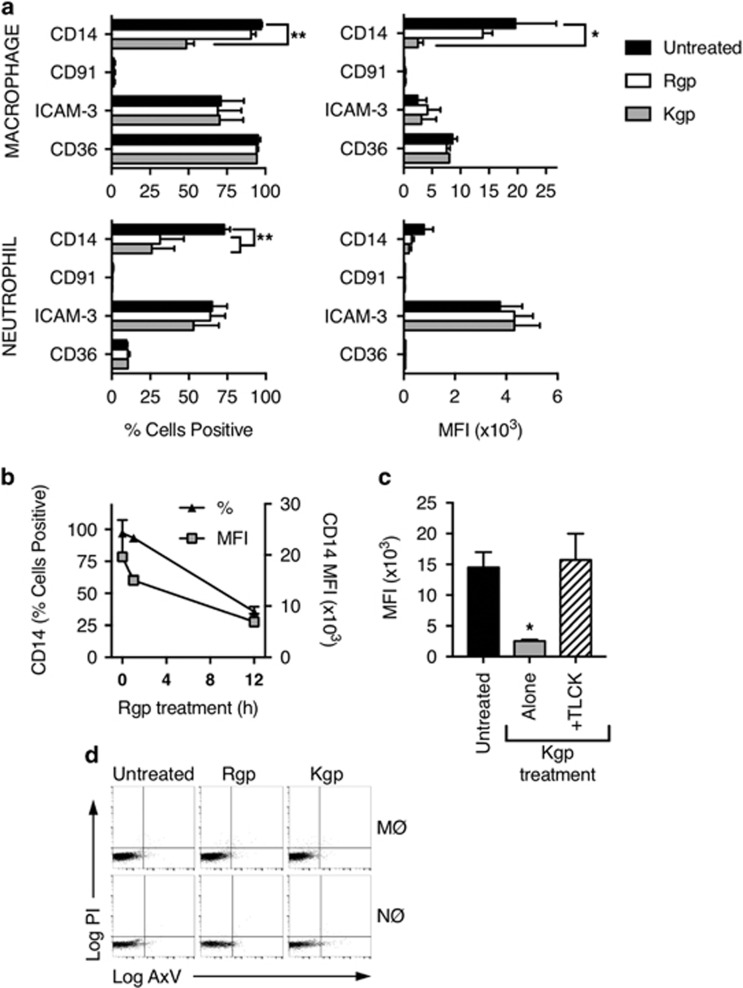
Purified gingipains cleave CD14 from the surface of macrophages and NØ. (**a**) Human primary NØ or THP-1 cell-derived MØ were treated with the indicated purified gingipains for 1 h at 37 °C prior to indirect immunofluorescence staining for receptors/ligands implicated in apoptotic cell clearance. Fluorescence levels are shown as percentage of cells positive for antigen (left) and mean fluorescence intensity (right). (**b**) THP-1 cell-derived MØ were treated with Rgp for up to 12 h at 37 °C prior to indirect immunofluorescence staining for CD14 at the indicated times. Fluorescence levels are shown as percentage of cells positive for antigen and mean fluorescence intensity. (**c**) MØ were treated with Kgp for 1 h in the presence or absence of the inhibitor TLCK prior to assessment of CD14 expression via flow cytometry. (**d**) Representative flow cytometric analyses of THP-1-derived MØ or NØ treated with RgpB or Kgp for 1 h at 37 °C and stained with annexin V and propidium iodide to reveal cell viability. Flow data shown are collected from at least 5000 events. Data presented are mean±S.E.M. of at least three independent experiments. Statistical analysis was performed using ANOVA followed by a Bonferroni *post hoc-*test. **P<0.01; ***P<0.0001 compared with untreated cells

THP-1 cells treated with dihydroxyvitamin D3 (VD3) were used as a model phagocyte, expressing high levels of CD14, that is functional for the clearance of AC.^5, 24^ MØ or primary human neutrophils (NØ) were treated with purified RgpB or Kgp and the expression of a panel of molecules (CD14, CD36, ICAM-3 and CD91) known to mediate AC clearance was assessed using indirect immunofluorescence and flow cytometry (Figure 2a). Kgp specifically reduced the expression of CD14, but not ICAM-3 or CD36, on the surface of both MØ and NØ with 1 h of treatment. However, while Rgp also specifically reduced CD14 expression on NØ with 1 h treatment, an extended period of incubation was required to see the same reduction on MØ (Figure 2b), a reduction that was not associated with a loss of MØ viability (Supplementary Figure 1). In addition, the rapid reduction in CD14 seen on Kgp-treated MØ was rescued using the specific inhibitor TLCK (Figure 2c), suggesting gingipains cleave CD14. Crucially, such gingipain treatment of MØ did not induce detectable cytotoxicity (Figure 2d). These data suggest that gingipains are capable of specific cleavage of the apoptotic cell tethering receptor CD14, data in agreement with previous reports.^16, 26, 27^

### Gingipains inhibit the directional migration of phagocytes to AC

As previous results demonstrated specific and rapid cleavage of CD14 by gingipains, we subsequently assessed the impact of this on effective clearance of AC. Here we assessed the impact of gingipain treatment on both the migration of phagocytes towards dying cells and the removal of cell corpses.

**Figure 3:**
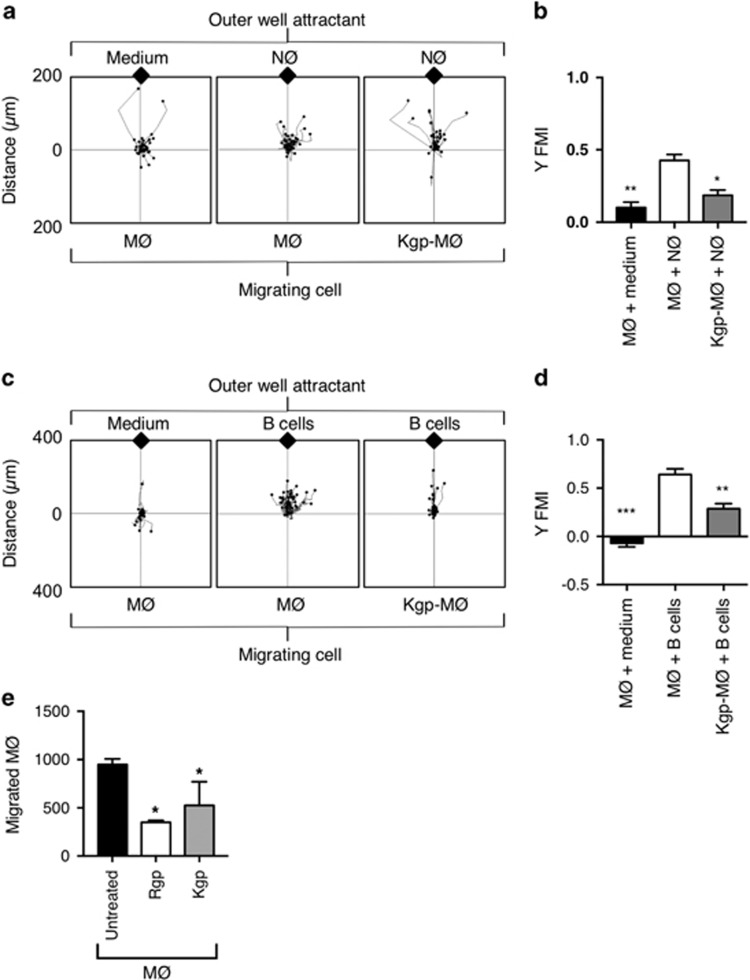
Gingipains inhibit macrophage migration towards AC. THP-1 cell-derived MØ were exposed to putative attractants in a horizontal or vertical migration assay. For the horizontal assay (**a**–**d**), MØ were seeded to glass coverslips and treated or untreated with gingipains before loading to a Dunn horizontal chamber and exposed to putative attractants (**a**–**d**). MØ migration was monitored for 2 h at 37 °C using time-lapse video microscopy. Migration of 40 cells per assay was measured using ImageJ and Ibidi Chemotaxis and Migration Tool (V2.0). The MØ route of travel is shown by a line from the starting point (set at the cross hairs of the plots) to the final MØ position after 2 h (black dot). The relative location of AC was at the top of the plot (black diamond). (**a**) Representative plots showing: left panel: untreated MØ (MØ) migration with control, cell-free medium as putative attractant to reveal basal levels of MØ migration. Centre panel: untreated MØ (MØ) migration with apoptotic NØ as attractant. Right panel: Kgp-treated MØ (Kgp-MØ) migration with apoptotic NØ as attractant. (**b**) Forward migration index (Y FMI) to quantify MØ migration in the direction of the putative gradient. (**c**) Representative plots showing: left panel: untreated MØ (MØ) migration with control, cell-free medium as putative attractant to reveal basal levels of MØ migration. Centre panel: untreated MØ (MØ) migration with apoptotic B cells as attractant. Right panel: Kgp-treated MØ (Kgp-MØ) migration with apoptotic B cells as attractant. (**d**) Forward migration index (Y FMI) to quantify MØ migration in the direction of the putative gradient. (**e**) For the vertical migration assay, MØ (untreated or treated with gingipain) were seeded to a transwell above a lower well containing secretome from apoptotic NØ and MØ migration to lower chamber assessed after 10 h by cell counting. Data shown in (**b** and **d**) are representative of four distinct experiments with two replicates in each experiment. Statistical analysis was conducted using ANOVA followed by Bonferroni *post hoc*-test *P< 0.05, **P<0.01, ***P< 0.001

To consider the effect of gingipains on MØ migration, THP-1 MØ were treated initially with Kgp as it acts rapidly on MØ receptors (Figure 2a) and then exposed to AC cultures (primary NØ or Burkitt's lymphoma (BL) cells) in a horizontal Dunn chemotaxis chamber system. Previously, we have shown that apoptotic BL cells serve as a relevant model to study the binding and uptake by phagocytes^5, 22^ and hence were used as a positive control. Phagocyte migration was recorded for 2 h at 37 °C using time-lapse video microscopy (Figure 3). Phagocytes were clearly motile throughout the assay period even in the absence of a putative attractant (left panel, Figures 3a and c). However, when phagocytes were exposed to apoptotic NØ (middle panel, Figure 3a) or apoptotic B cells (middle panel, Figure 3c), rapid and directional movement of MØ was evident. Kgp-treated MØ continued to migrate, however, gingipain-treated phagocyte migration towards AC was significantly reduced (right panel, Figures 3a and c). This was shown by a reduced forward migration index for both apoptotic BL cells and primary apoptotic NØ compared with untreated MØ migration towards the AC (Figures 3b and d). Importantly, other measures of migration (specifically velocity, accumulated distance and Euclidean distance) were unaffected (Supplementary Figure 2). Thus, treatment of MØ with Kgp, significantly reduced only the directionality of MØ migration towards dying cells, impairing effective MØ recruitment to dying cells. Further studies employed a vertical migration system to address further MØ migration following gingipain treatment. These studies demonstrate that Rgp treatment also inhibits MØ migration to apoptotic NØ (Figure 3e).

### Gingipains inhibit the interaction of AC with MØ

Inefficient removal of apoptotic NØ may result in significant periodontal tissue damage if they are not cleared rapidly by MØ.^28^

**Figure 4:**
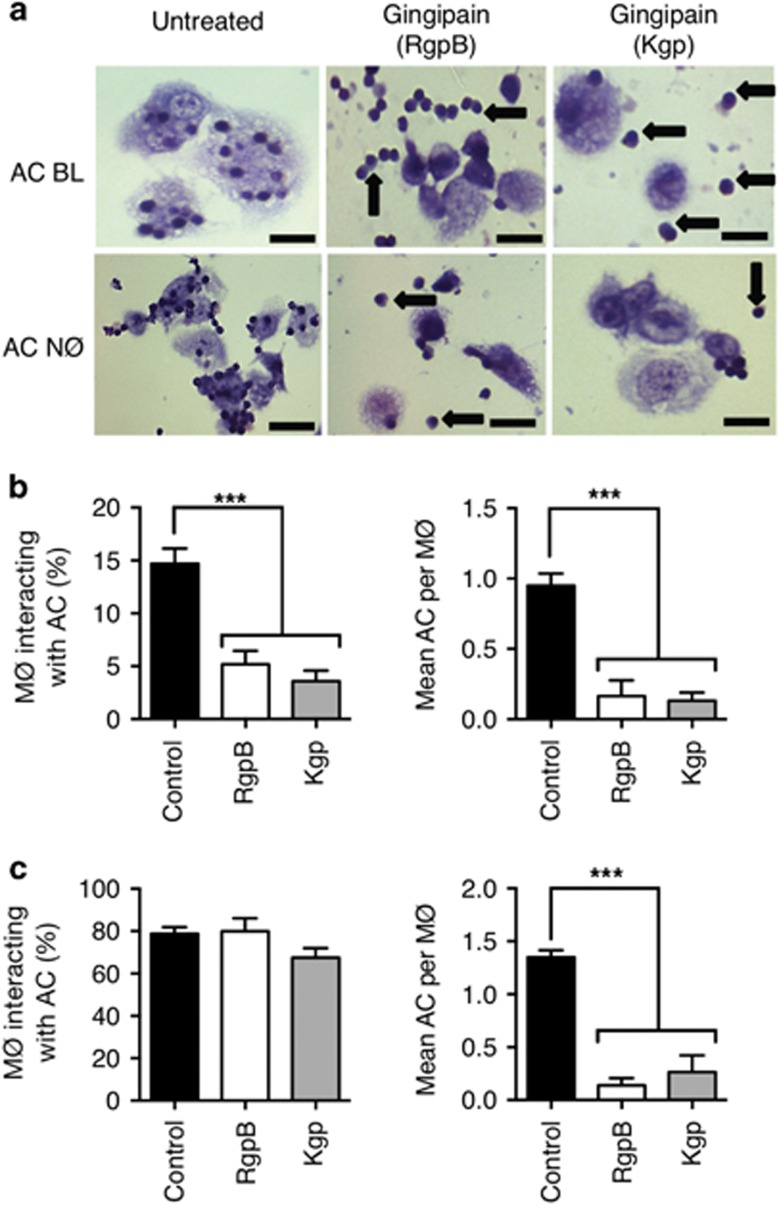
Gingipain treatment of MØ inhibits interaction of MØ with apoptotic NØ and apoptotic BL cells. THP-1-derived MØ were treated or untreated with gingipains prior to co-culture with AC for 1 h. (**a**) Photomicrographs of Jenner–Giemsa stained untreated or gingipain-treated THP-1 cell-derived MØ interacting with apoptotic BL cells (AC BL) or primary apoptotic NØ (AC NØ). Untreated THP-1 MØ showing tethering and phagocytosing of apoptotic BL (upper left panel) and NØ (lower left panel). Gingipain-treated THP-1 MØ (Rgp: centre panels; Kgp: right panels) showing interaction with fewer apoptotic BL cells (upper panels) and NØ (lower panels) with an increased number of unbound/non-phagocytosed, extracellular AC (arrows). THP-1 MØ can be seen in light blue and AC are stained a characteristic intense blue. Arrows in the lower right panel indicate primary apoptotic NØ not taken up by phagocytes. All images were taken at × 40 magnification. (**b**) Histograms showing the percentage of MØ interacting (binding or phagocytosing) with apoptotic NØ cells (left panel) or the mean number of AC per MØ (right panel) with or without gingipain treatment of MØ or (**c**) histograms showing the percentage of MØ interacting (binding or phagocytosing) with apoptotic BL cells (left panel) or the mean number of AC per MØ (right panel) with or without gingipain treatment of MØ. Data shown are mean±S.E.M. of at least three independent experiments. Statistical analysis was conducted using ANOVA followed by Bonferroni *post hoc*-test: ***P<0.001

Previous work has demonstrated the importance of CD14 as a tethering receptor for AC through the use of anti-CD14 mAbs^22, 23^ or CD14-deficient animals.^29^ These studies demonstrated that MØ CD14 binds to, as yet unidentified, ligands at the surface of a wide range of AC. Given the gingipain-mediated reduction in CD14 expression (Figure 2), the impact of gingipain treatment on AC removal was assessed. THP-1 MØ were co-cultured with AC (BL or NØ) and the level of AC–MØ interaction scored and compared with that of equivalent gingipain-treated MØ. Photomicroscopy revealed evidence of MØ interacting with AC (Figure 4a), where ‘Interaction' is defined as either binding or phagocytosed AC, irrespective of the number of AC bound or internalised. When this level of interaction was scored, it was clear that the percentage of MØ interacting with apoptotic NØ cells was significantly reduced following gingipain treatment of MØ (left panel, Figure 4b). This effect was more evident when scoring the mean number of AC per MØ (right panel, Figure 4b). However, when apoptotic BL cells were used, gingipains did not cause a decrease in interaction (left panel, Figure 4c), a consequence of their failure to inhibit completely the interaction of apoptotic BL cells with MØ. A clear gingipain inhibitory effect was however noted when the mean number of AC per MØ was scored (right panel, Figure 4c). In these assays, both gingipains assayed reduced AC clearance indicating that this effect is not mediated solely through the reduced expression of CD14 as, in this time frame, only Kgp reduced MØ CD14 surface levels. Importantly, gingipain treatment had no significant effect on MØ viability (Supplementary Figure 1).

### TNF-*α* production by MØ in response to *P. gingivalis* LPS is inhibited by AC or their derived secretomes

**Figure 5:**
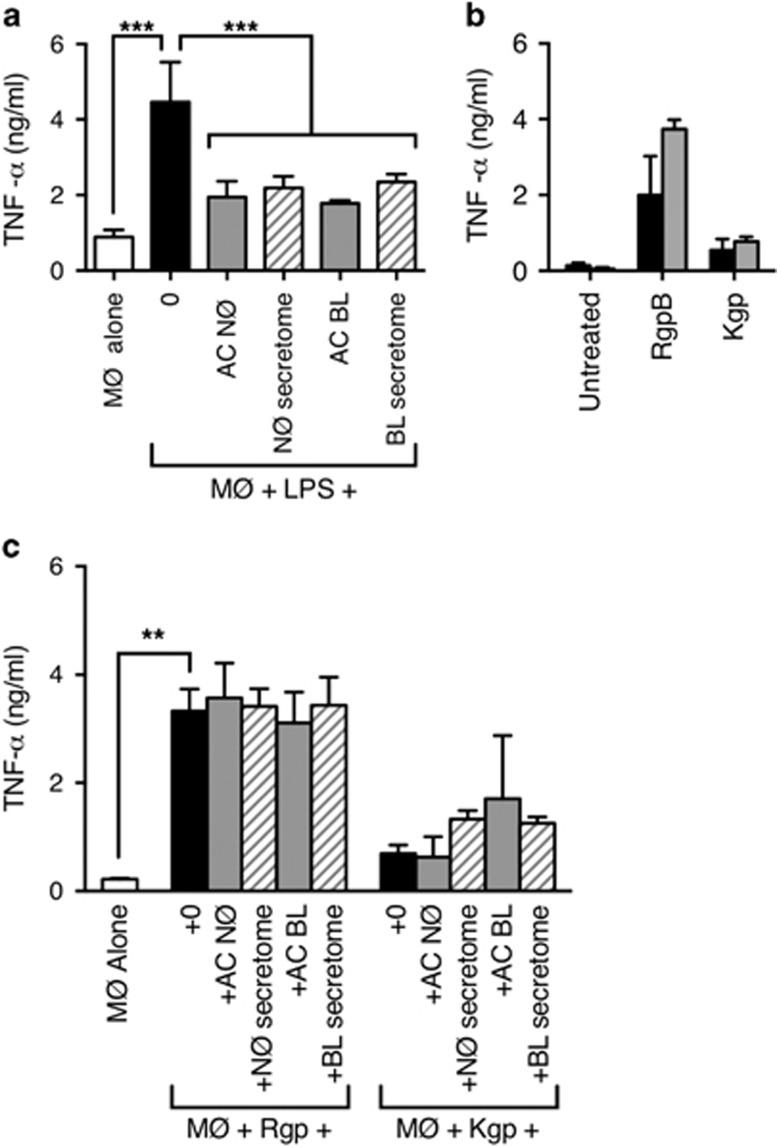
Apoptotic NØ inhibit LPS-induced but not gingipain-induced TNF-*α* production (**a**) THP-1-derived MØ (VD3/PMA) were co-cultured with AC (NØ or BL) or their derived secretomes for 18 h prior to the addition of LPS from *P. gingivalis*. After 4 h stimulation, TNF-*α* production was assessed using ELISA. (**b**) THP-1-derived MØ (black: VD3-differentiated THP-1; grey: VD3/PMA-differentiated THP-1) were treated with the indicated gingipain for 1 h immediately prior to ELISA for produced TNF-*α*. (**c**) THP-1-derived MØ (VD3/PMA) were co-cultured with AC (NØ or BL) or their derived secretomes for 18 h prior to treated with gingipain from *P. gingivalis* to ELISA for produced TNF-*α*. Data shown are mean±S.E.M. for three independent experiments. Statistical analysis was conducted using ANOVA followed by Bonferroni *post hoc*-test: **P<0.01, ***P<0.001

AC clearance is known for its non-phlogistic phenotype and AC have been reported widely to exert potent anti-inflammatory effects to promote resolution of inflammation.^30, 31, 32^ To explore the nature of the inflammatory responses of MØ in the presence of AC, MØ production of TNF-*α* in response to LPS from *P. gingivalis* was assessed in the presence or absence of apoptotic NØ or apoptotic BL cells. Additionally, AC are known to release a range of factors (the secretome), including apoptotic cell-derived extracellular vesicles, into their supernatant that can modulate apoptotic cell clearance.^3, 5, 6, 33^ *P. gingivalis* LPS was capable of inducing strong TNF-*α* production that was inhibited following MØ incubation with either AC or their derived secretome (Figure 5a). This suggests that it is possible for AC to exert a dominant anti-inflammatory effect on TNF-*α* production induced by the pathogen *P. gingivalis*.

### Gingipains induce TNF-*α* production by MØ that is not inhibited by AC

When considering the effects of gingipains on the ability of AC to modulate inflammation, we noted that gingipain treatment alone of MØ was sufficient to induce TNF-*α* production in just 1 h (Figure 5b). This effect was observed with two different models of THP-1-derived MØ (VD3 or PMA/VD3) that are known to have profoundly different sensitivity to LPS (Supplementary Figure 3).^24^ Notably, the responses of these cells to gingipain preparations were similar, which indicates that the detected inflammatory response was not due to LPS contamination of gingipain preparations. This observation raises the possibility that chronic inflammation may derive from the direct action of gingipains on pro-inflammatory responses and not simply through indirect effects (e.g., through inhibited AC removal).

Given this intrinsic induction of inflammation, we were interested to consider the ability of AC or their derived secretome to modulate this response. In line with the previous experiments (Figure 5a), MØ were treated with AC or secretome prior to addition of the inflammatory stimulant (Rgp or Kgp) and analysis of TNF-*α* production. Although AC or their derived secretome inhibited LPS-induced TNF-*α* production (Figure 5a), these cells or their secretomes had no ability to reduce gingipain-induced inflammation (Figure 5c). These data raise the possibility that gingipains induce local tissue inflammation that is resistant to the usual mechanisms that aim to promote the resolution of inflammation.

Taken together, the data presented here support the notion that the key gingival pathogen *P. gingivalis* promotes inflammation by different mechanisms. These include inhibition of apoptotic cell clearance via the action of gingipains on apoptotic cell clearance mechanisms including CD14 and through the direct gingipain induction of inflammation that is recalcitrant to the usual inhibitory effects of apoptosis.

## Discussion

Previous studies have shown that a major factor in periodontal disease is the significant proteolytic activity of *P. gingivalis* including the three gene products encoding gingipains.^26, 34, 35^ In the present study, we characterise two gingipains (Kgp and Rgp) for their ability to modulate a key event in the resolution of inflammation, that is, the rapid clearance of AC. For the first time, we take a more complete approach to the *in vitro* analysis of AC clearance and consider the impact of gingipains not only on the binding and phagocytosis of AC but also on the migration of phagocytes towards cell corpses and their immune modulation by dying cells. Migration of phagocytes to AC is a key event in the clearance process that is often overlooked in *in vitro* assays of AC removal.

Here we report isolation of Kgp and Rgp from *P. gingivalis* using established methods. Our analyses of protease activity are consistent with successful isolation in line with previous work.^20, 21, 34, 36^

Periodontal disease is a chronic, non-resolving inflammatory condition characterised by persistent neutrophilic activity. During acute inflammation, neutrophil recruitment is followed by neutrophil apoptosis and rapid non-phlogistic clearance of AC by MØ. The course of AC clearance is a complex, multistage process that begins with AC communicating their presence via their secretome (i.e., apoptotic cell-derived extracellular vesicles and soluble factors), which act as ‘find me' signals to phagocytes (reviewed in ref. 37). Following phagocyte recruitment, an array of ligands, receptors and bridging molecules effect binding and removal of cell corpses (reviewed in ref. 37). Additionally, immune modulation of the microenvironment supports pro-resolution and anti-inflammatory effects.^38^ Given the importance of AC clearance to the control of inflammation, we sought to define the impact of gingipains on these processes to understand better the defects that may contribute to periodontal disease.

The recruitment of phagocytes to sites of cell death is an essential process to promote the effective resolution of inflammation. Here we show that MØ treated with gingipains are less direct in their migration towards dying cells. Although our data suggest a correlation between CD14 loss and inhibited migration following protease treatment, this does not identify formally a causal relationship. Indeed, Rgp profoundly inhibited MØ migration at time points when CD14 cleavage was low. Thus, the precise molecular mechanism by which this gingipain-mediated inhibition is effected remains to be fully elucidated, however, it remains a possibility that this is mediated through CD14. Previous work^33^ suggests CD14 is not required for ‘sensing' dying cells though the study did not assess directionality as was studied here. Additionally, recent reports of CD14-mediated leukocyte migration^39, 40^ suggest the CD14 role in macrophage migration towards AC deserves close attention. Further studies are required to define alternative mechanisms of action, possibly mediated through GPCR chemokine receptors, for example, proteinases from *P. gingivalis* cleave the human C5a receptor.^41^ Thus CX_3_CR_1_, which promotes migration to AC, or cleavage of receptors for secretome factors (e.g., apoptotic cell-derived extracellular vesicles) that also promote migration^3, 5, 6, 33^ may be likely targets for gingipains. Irrespective of the mechanism, any reduction in directional migration is likely to impact on the course of inflammation in periodontitis. This may also be a mechanism by which systemic effects of gingipains occur, with poorly controlled MØ recruitment throughout the body.

Here we demonstrate gingipains cleave MØ CD14 in line with previous studies.^16, 26^ Treatment with LPS alone had no such effect. We demonstrate that Kgp cleaves CD14 more rapidly than Rgp, in agreement with previous observations of relative activity of gingipains.^42^ This may be due to the presence of a hemagglutinin/adhesion domain present in Kgp but not RgpB.^20^ Our data correlate with previous clinical research reporting less CD14 expression in chronic periodontitis samples than in healthy periodontal gingival samples, supporting the notion that CD14 expression correlates with periodontal health.^43^ Such cleavage within 1 h of protease exposure raises the potential for rapid onset of detrimental effects associated with CD14 loss. Additionally, it is highly likely that other receptors may be cleaved though we detected no reduction of ICAM-3, CD36 or CD91. This is surprising, given the presence of putative gingipain cleavage sites within the protein sequences but highlights that accessibility of cleavage sites may be key in determining whether any given molecular target can be cleaved. Interestingly, we demonstrate also that neutrophil CD14 is cleaved suggesting ligands on dying leukocytes may be affected in the periodontal microenvironment. Previous work suggests that loss of the CD31 from NØ treated with Rgp but not Kgp can promote NØ removal by MØ.^19^ Although we demonstrate CD14 cleavage, it is likely other ligand/receptors are also cleaved and a full assessment of all molecular targets would be worthwhile.

The mechanisms by which dying cells are removed exhibit a high level of redundancy.^44^ However, CD14 is a key tethering receptor for AC^22^ that is non-redundant as CD14-deficient animals carry an increase in persistent cell corpses.^29^ Interestingly, Truman *et al.*^33^ suggested that MØ CD14 may be upregulated during migration towards AC as they become increasingly competent to remove cell corpses. Taken together, it seems likely that the rapid cleavage of CD14 and consequent failure for efficient AC clearance will promote defective control of inflammation in periodontitis. Cell persistence of hyperactive NØ from the gingival crevice of periodontitis patients will drive collateral tissue damage^45^ and failed removal of activated NØ may cause tissue destruction as they undergo secondary necrosis, leading to periodontal tissue damage.^28^ Our research correlates also with previous studies highlighting the level of necrotic cell death, likely as a result of failed clearance, in NØ^46, 47^ in addition to their release of destructive hydrolytic enzymes^48, 49, 50^ found in gingival crevicular fluid from the active phase of periodontitis. These support the notion that failure to uptake apoptotic NØ following gingipain cleavage of specific receptors, for example, CD14, will drive necrosis resulting in connective tissue matrix destruction in periodontitis.

It is also noteworthy that previous studies have also shown that CD14 cleavage, via human neutrophil elastase, can inhibit clearance of AC^51^ though it was the potential for protease inhibition, via delivery of inhibitors, to recover effective AC that provides a clear therapeutic rationale within periodontal disease. Furthermore, this previous work highlights the likely significance of CD14 cleavage on phagocyte cellular function.

The balance of pro-inflammatory and pro-resolution/anti-inflammatory cellular responses will decide the nature and duration of an inflammatory response in the tissue. It is well established that AC exert strong anti-inflammatory effects^30, 31^ and that the onset of inflammation initiates events that ultimately promote resolution.^38^ Here we assessed the ability of apoptotic human NØ and their derived secretome to inhibit inflammation induced by *P. gingivalis* LPS. In line with previous reports using *E. coli* LPS,^24, 30, 31, 32^ we noted a strong anti-inflammatory effect suggesting dying cells, within a periodontium infected with *P. gingivalis*, ought to be capable of promoting resolution of inflammation. However, we noted that gingipain treatment of MØ alone was sufficient to induce inflammatory responses. Recently, gingipains have been reported to promote monocyte inflammatory responses^52^ via protease-activated receptors and Toll-like receptors/NOD-like receptor ligation.^53^ Furthermore, gingipains have been shown to cleave protease-activated receptors on oral keratinocytes resulting in innate immune responses.^54^ Kgp is capable of generating a keratin fragment from epithelial cells, which induces inflammatory responses in epithelial and fibroblast cells.^55^ Importantly, this gingipain-induced inflammation was not modulated by AC suggesting that gingipain-induced inflammation may be dominant within the periodontal microenvironment, possibly as a result of loss of key immune modulatory receptors and/or induction of potent inflammatory responses. This may be responsible for driving a continued inflammatory response.

Although our study has focused on the impact of gingipains on the sensing and handling of dying leukocytes, it is important to note that CD14 is an innate immune receptor and the modulation of CD14, and other such receptors, by gingipains is likely an immune evasion strategy of the pathogen.^16^ Thus, when considering the complete periodontal environment, one should also assess the impact of gingipain treatment on phagocyte migration towards *P. gingivalis* and subsequent immune clearance.

Taken together, we propose that gingipains can have profound and varied effects on the resolution of inflammation. The inhibition in directional migration of MØ towards dying cells, coupled with reduced efficiency of apoptotic cell clearance and induction of dominant pro-inflammatory responses by gingipains, will promote the chronic inflammatory milieu characteristic of the progressive and debilitating periodontal disease. Thus, we support gingipains as an attractive target for therapeutic intervention.

## Materials and Methods

### Cell isolation, cell lines and culture

Peripheral blood NØ were collected from healthy donors using EDTA - BD vacutainers and isolated using a two-step discontinuous Percoll column (1.079 and 1.098 g/ml) with centrifugation (150 × *g* for 8 min, followed by 1200 × *g* for 10 min without braking). Isolated NØ were washed and resuspended in PBS supplemented with 1.5% (w/v) glucose and cations (1 mM MgCl_2_, 1.5 mM CaCl_2_) prior to assessment of NØ purity or in RPMI 1640 medium containing 0.15% (w/v) BSA (‘NØ medium') for generation of AC. Human monocytic leukaemia THP-1 and EBV-positive BL.^56^ Mutu cells were cultured in ‘complete' RPMI 1640 (RPMI containing 10% (v/v) foetal calf serum, 2 mM L-glutamine, 100 IU/ml penicillin, 100 μg/ml streptomycin (PAA Laboratories Ltd Yeovil, Somerset, UK)). The cells were cultured at 37 °C in a humidified environment at 5% (v/v) CO_2_.

THP-derived macrophage-like cells were produced by differentiation over 48–72 h with VD3 (100 nM) or both VD3 and 250 nM phorbol ester (PMA; Sigma, Dorset, UK) as described previously.^24^ Where appropriate, MØ were treated with gingipain (5 *μ*g/ml of purified isolated enzyme) in serum-free RPMI medium for 1 h at 37 °C prior to addition of complete RPMI and further analyses.

### Bacterial strains and growth conditions

*P. gingivalis* ATCC 33277, W83 and HG66 was kindly provided by the Periodontal Research Group, School of Dentistry, University of Birmingham, UK. Bacteria were grown in liquid medium (Fastidious Anaerobe Broth, LAB M, Lancashire, UK) containing 5% sheep blood agar containing, per litre, 10.0 g yeast extract, 5 mg haemin, 0.5 mg vitamin K1, 500 mg L-cysteine were incubated at 37 °C in an anaerobic atmosphere (miniMACS anaerobic workstation; Don Whitley Scientific, UK) of 10% H_2_, 10% CO_2_ and 80% N_2_. Growth of the *P. gingivalis* strains was monitored spectrophotometrically over a period of 72 h (optical density at 600 nm) and samples of the cultures were Gram stained to confirm their purity.

### Isolation, purification and identification of gingipains

Gingipains from HG66 were isolated according to the method described by Pike *et al.*^21^ The crude extract of outer membrane fractions from *P. gingivalis* strain W83 were prepared according to ref. 57. Enzyme activity and molecular weight of the purified proteins were determined as described by Pike *et al.*^21^

Following bottom-up proteomics approach,^58^ purified gingipains were positively identified using LC-MS/MS (QExactive ThermoFisher, Rugby, UK). Acquired data were searched against SwissProt database using Sequest search engine. For both gingipains, more than 30 peptides with at least two unique sequences were identified, thereby assuring at least 28% of total protein coverage and providing the highest identification scores (Score >1000) (Table 1 in Supplementary Material).

### Apoptosis induction and quantification

Human primary NØ and the human BL cell line (Mutu) were induced to apoptosis either by UV-B irradiation using a Chromata-vue C71 light box and UVX radiometer (UV-P Inc, Uplands, CA, USA) or exposure to proteolytic enzymes from *P. gingivalis*. BL cells were resuspended in complete RPMI medium and exposed to 50 mJ/cm^2^. Peripheral blood neutrophils resuspended in 0.15% (w/v) bovine serum albumin (Sigma-Aldrich, Poole, Dorset, UK), 100 μg/ml penicillin and 100 mg/ml streptomycin were cultured at 37 °C for 16 h and used as a source of AC. Where apoptotic cell-secretome was used, apoptotic cell cultures were centrifuged at 2000 × *g* for 20 min to remove cell and large debris and the supernatant was used. Apoptosis was quantified using annexin V-FITC and propidium iodide (eBioscience, High Wycombe, UK) staining according to the manufacturer's instructions. Briefly, cells (5 × 10^5^–1 × 10^6^) were washed with ‘binding buffer' (10 mM HEPES, 150 mM NaCl and 2.5 mM CaCl_2_ in water) and resuspended in ‘binding buffer' containing annexin V (1 *μ*l per 200 000 cells) and incubated on ice for 5 min. Incubated cells were either washed with buffer and/or added directly to propidium iodide to a final concentration of 20 *μ*g/ml and analysed immediately by flow cytometry (FC500; Beckman Coulter, Fullerton, CA, USA).

### Surface receptor expression on MØ and NØ

THP-1 cells differentiated with VD3 for 48–72 h were exposed to purified gingipains RgpB or Kgp (from strain HG66) at various concentrations and time periods. Cells were washed with PBS containing 1% (w/v) BSA and stained with anti-mouse CD14 (clone 63D3) followed by anti-mouse IgG FITC (Sigma-Aldrich) and receptor expression was determined by flow cytometry. The percentage of cells positive for antigen and the mean fluorescence intensity were analysed using FlowJo analysis software (FlowJo, OR, USA).

### Horizontal migration assay

The phagocytic cells used were 48 h THP-1/VD3 differentiated MØ cultured on a coverslip in a six-well plate. DUNN chamber glass slides (Hawskley, Lancing, Sussex, UK. DCC100) were used according to the manufacturer's instructions. Briefly, a coverslip carrying THP-1-derived MØ was placed on the chamber containing macrophage medium (Invitrogen Corp., Paisley, UK) and 100 *μ*l of chemoattractant was loaded to the outer well before the chamber was sealed with wax. The cell behaviour was recorded at 37 °C for 2 h using a fully motorised Zeiss Axiovert 200M fluorescence microscope (Carl Zeiss Ltd., Welwyn Garden City, UK) and images collected every 10 min by a Hamamatsu Orca camera driven by Volocity (Perkin-Elmer, Cambridge, UK). Cell tracking was evaluated using ImageJ followed by the chemotaxis and migration tool software (version 2.0; Ibidi GmbH, Martinsried, Germany).

### Vertical migration assay

MØ migration towards AC was assessed using the Cell-IQ tracking system (CM Technologies, Tampere, Finland). THP-1 stimulated with VD3 (48 h) were used as phagocytes. To set up the experiment, 700 *μ*l per well of AC (1 × 10^6^) or their derived secretomes were added to the wells of a 24-well transwell companion plate (Falcon, Corning, Amsterdam, the Netherlands). A transwell with 8.0 *μ*m pore PET membranes (Falcon, Corning) was subsequently placed in each well 300 *μ*l of MØ (5 × 10^5^) were added. Migration of MØ from the upper transwell chamber to the lower well was recorded for 10 h using the Cell-IQ system. Migrated cells were detected by their appearance on the base of the lower well. Data were analysed using the Cell-IQ software.

### Assay of apoptotic cell–MØ interaction

THP-1-derived MØ (THP-1/PMA) were cultured on four-well glass slides (Hendley (Essex) Ltd, Loughton, UK). AC were induced to apoptosis and after overnight incubation were washed via centrifugation into fresh RPMI containing 0.2% w/v BSA (Sigma-Aldrich). AC were co-cultured with MØ at a ratio of 100 : 1 for 1 h at 37 °C in RPMI containing 0.2% w/v BSA (Sigma). Unbound cells were removed by washing in cold PBS and slides were fixed in methanol prior to staining with Jenner–Giemsa. Interaction was scored as the percentage of MØ that had AC associated, where the AC could be surface bound or internalised. Where appropriate, the mean number of AC per MØ was also enumerated.

### Measurement of TNF-*α*

TNF-*α* levels were measured in supernatants of macrophage exposed to purified gingipains incubated for 1 h at 37 °C. Subsequently, the reaction was inactivated by washing the cells with PBS containing 1% (w/v) BSA prior to challenge with LPS from *P. gingivalis* (InvivoGen, San Diego, CA, USA) for 4 h in the presence of AC. TNF-*α* was determined by ELISA using matched-pair capture and biotinylated detection antibodies (R&D Systems, Abingdon, UK). Bound detection Ab was detected using streptavidin-HRP followed by colorimetric SigmaFAST OPD assay (Sigma).

### Statistical analysis

Data were analysed using Graphpad (La Jolla, CA, USA) from at least three independent experiments unless otherwise stated and presented as the mean±S.E.M.
