# Supplementary figures and images for: Porphyromonas gingivalis gingipains cause defective macrophage migration towards apoptotic cells and inhibit phagocytosis of primary apoptotic neutrophils
Source: Cell Death Dis. 2017 Mar 2;8(3):e2644–. doi: 10.1038/cddis.2016.481 (PMC5386511; doi:10.1038/cddis.2016.481)

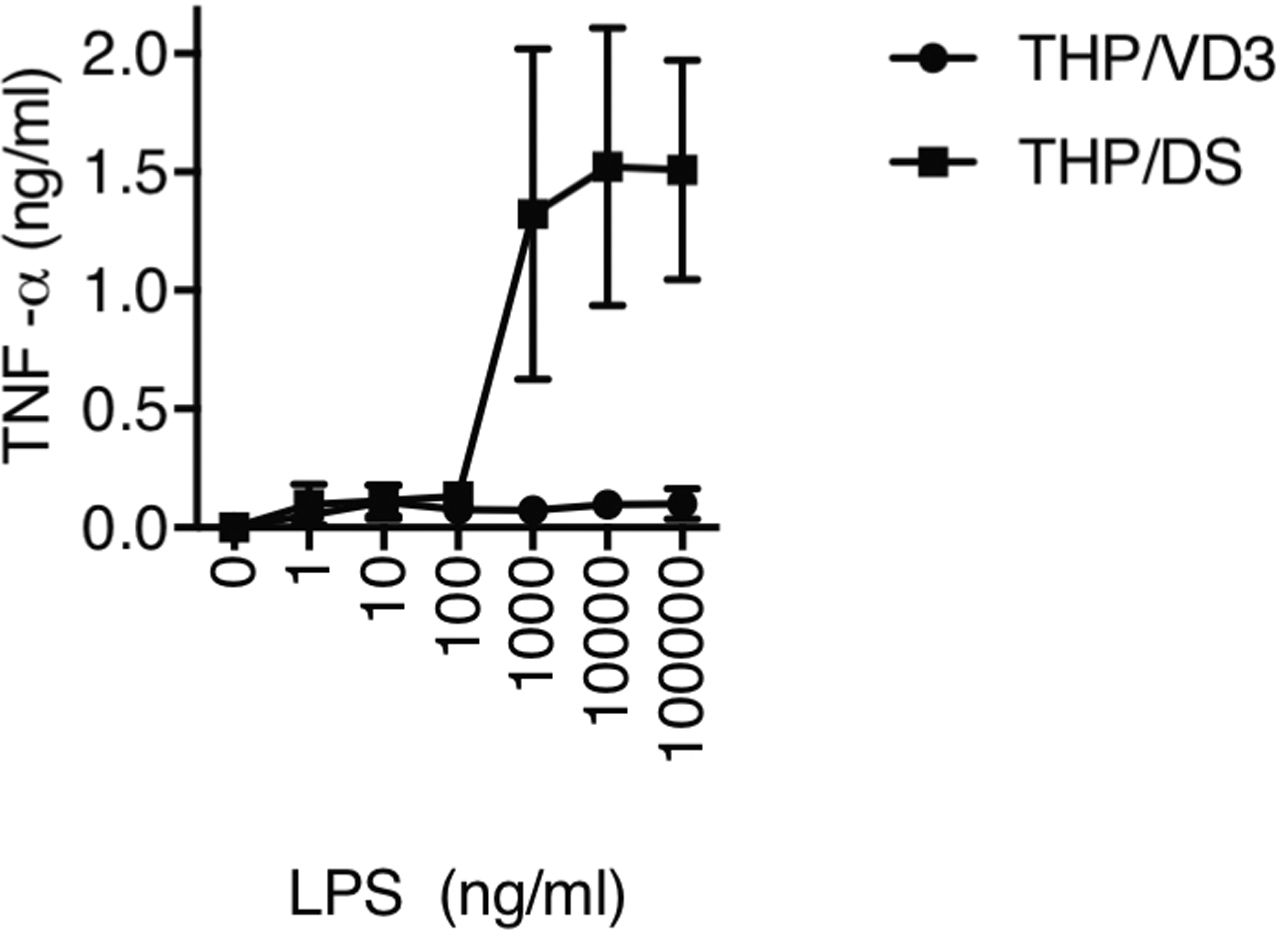

Supplement: Supplementary Figure 3 [file cddis2016481x3.tif]
