# Supplementary material for: Porphyromonas gingivalis gingipains cause defective macrophage migration towards apoptotic cells and inhibit phagocytosis of primary apoptotic neutrophils
Source: Cell Death Dis. 2017 Mar 2;8(3):e2644–. doi: 10.1038/cddis.2016.481 (PMC5386511; doi:10.1038/cddis.2016.481)
Supplement: Supplementary Information [file cddis2016481x4.doc]

**SUPPLEMENTARY TITLES AND LEGENDS TO FIGURES & TABLE 1**

**Supplementary Figure 1: Extended treatment of macrophages with gingipain does not affect cell viability, size or granularity.** THP-1 cell-derived MØ were treated with Rgp, Kgp or left untreated for 16h. MØ were subsequently stained with annexin V to assess cell viability via flow cytometry. (A) The percentage of cells negative for annexin V (i.e. viable) are shown for three independent experiment. (B) Representative forward scatter (FS) and side-scatter (SS) plots are shown to reveal MØ size and granularity respectively. (C) FS and SS measures for replicate experiments. Data shown are mean ± SEM for three independent experiments. Statistical analysis was conducted using ANOVA followed by Bonferroni post-test: ns.

**Supplementary Figure 2: Treatment of macrophages with Kgp gingipain does not affect MØ migration distance or velocity.** THP-1 cell-derived macrophages (untreated or Kgp-treated) were exposed to putative attractants in a horizontal Dunn chamber assay. MØ migration was monitored for 2 h at 37°C using time-lapse video microscopy. Migration of 40 cells per assay was measured using Image J and Ibidi Chemotaxis and Migration Tool (V2.0). (A) Data shown are measures of MØ migration towards apoptotic BL cells: Euclidian Distance, Accumulated Distance and Velocity. (B) Data shown are measures of MØ migration towards apoptotic neutrophils: Euclidian Distance, Accumulated Distance and Velocity. Data shown mean±SEM for at least 3 independent replicates. Statistical analysis was conducted using ANOVA followed by Bonferroni post-test ns=not significant.

**Supplementary Figure 3: LPS from *P.gingivalis* induces a dose-dependent production of TNF-α in THP-1 cell-derived macrophages.** THP-1 cells were stimulated to differentiate with VD3 (THP/VD3) or double stimulated with VD3 and PMA (THP/DS) for 48h as indicated prior to treatment with the indicated concentrations of LPS for 4h prior to ELISA for produced TNF-α. Results shown are mean ± SEM for three independent experiments.

**Supplementary Table 1.** Proteins identified as a results of the Sequest search against SwissProt database, using *Porphyromonas gingivalis* taxonomy, trypsin as a proteolytic enzyme and allowing up to two miss-cleavages. Oxidation of the methionine residue, carbamidomethylation on the cystione thiol, and deamidation of asparagine and glutamine side chains were used as variable post-translational modifications. Only the high confidence, rank 1 and unique peptides were used for the identification, with at least two different sequences identified per protein.

Supplementary Table 1

| **Accession** | **Description** | **Score** | **Coverage** | **# Proteins** | **# Unique Peptides** | **# Peptides** | **MW [kDa]** |
| --- | --- | --- | --- | --- | --- | --- | --- |
| B2RM93 | Gingipain R1 OS=Porphyromonas gingivalis (strain ATCC 33277 / DSM 20709 / CIP 103683 / JCM 12257 / NCTC 11834 / 2561) GN=rgpA PE=3 SV=1 - [CPG1_PORG3] | 1833.17 | 37.29 | 2 | 3 | 52 | 185.2 |
| B2RKU0 | Gingipain R2 OS=Porphyromonas gingivalis (strain ATCC 33277 / DSM 20709 / CIP 103683 / JCM 12257 / NCTC 11834 / 2561) GN=rgpB PE=3 SV=1 - [CPG2_PORG3] | 1522.05 | 52.58 | 1 | 1 | 30 | 80.9 |
| P28784 | Gingipain R1 OS=Porphyromonas gingivalis GN=rgpA PE=1 SV=2 - [CPG1_PORGN] | 1448.00 | 45.21 | 1 | 1 | 38 | 108.7 |
| P95493 | Gingipain R2 OS=Porphyromonas gingivalis (strain ATCC BAA-308 / W83) GN=rgpB PE=1 SV=2 - [CPG2_PORGI] | 1206.98 | 34.92 | 1 | 1 | 23 | 80.9 |
| B2RLK2 | Lys-gingipain OS=Porphyromonas gingivalis (strain ATCC 33277 / DSM 20709 / CIP 103683 / JCM 12257 / NCTC 11834 / 2561) GN=kgp PE=1 SV=1 - [KGP_PORG3] | 1082.04 | 28.50 | 1 | 2 | 33 | 187.1 |
| P72197 | Lys-gingipain HG66 OS=Porphyromonas gingivalis GN=kgp PE=1 SV=1 - [KGP66_PORGN] | 1076.46 | 30.88 | 2 | 2 | 35 | 186.7 |
| Q9RQJ2 | Peptidylarginine deiminase OS=Porphyromonas gingivalis (strain ATCC BAA-308 / W83) GN=PG_1424 PE=1 SV=1 - [PAD_PORGI] | 344.40 | 68.35 | 1 | 29 | 29 | 61.7 |
| Q51845 | Hemagglutinin A OS=Porphyromonas gingivalis GN=hagA PE=3 SV=1 - [HAGA2_PORGN] | 146.89 | 14.80 | 1 | 6 | 10 | 283.1 |
| B2RH57 | Major fimbrium subunit FimC OS=Porphyromonas gingivalis (strain ATCC 33277 / DSM 20709 / CIP 103683 / JCM 12257 / NCTC 11834 / 2561) GN=fimC PE=1 SV=2 - [FIMC_PORG3] | 132.26 | 34.22 | 1 | 13 | 13 | 50.1 |
| B2RH59 | Major fimbrium tip subunit FimE OS=Porphyromonas gingivalis (strain ATCC 33277 / DSM 20709 / CIP 103683 / JCM 12257 / NCTC 11834 / 2561) GN=fimE PE=1 SV=1 - [FIME_PORG3] | 103.50 | 49.27 | 1 | 20 | 20 | 60.6 |
| B2RH54 | Major fimbrium subunit FimA type-1 OS=Porphyromonas gingivalis (strain ATCC 33277 / DSM 20709 / CIP 103683 / JCM 12257 / NCTC 11834 / 2561) GN=fimA PE=1 SV=3 - [FIMA1_PORG3] | 81.91 | 34.46 | 3 | 8 | 8 | 41.3 |
| P0C934 | NAD-specific glutamate dehydrogenase OS=Porphyromonas gingivalis (strain ATCC BAA-308 / W83) GN=gdh PE=1 SV=1 - [DHE2_PORGI] | 63.10 | 37.98 | 1 | 14 | 14 | 49.2 |
| B2RHG1 | Minor fimbrium subunit Mfa1 OS=Porphyromonas gingivalis (strain ATCC 33277 / DSM 20709 / CIP 103683 / JCM 12257 / NCTC 11834 / 2561) GN=mfa1 PE=1 SV=1 - [MFA1_PORG3] | 21.66 | 11.19 | 1 | 5 | 5 | 60.7 |
| Q9S3R8 | Outer membrane protein 40 OS=Porphyromonas gingivalis (strain ATCC BAA-308 / W83) GN=PG_0694 PE=1 SV=1 - [OMP40_PORGI] | 19.83 | 18.95 | 1 | 5 | 5 | 42.4 |
| B2RLL7 | Enolase OS=Porphyromonas gingivalis (strain ATCC 33277 / DSM 20709 / CIP 103683 / JCM 12257 / NCTC 11834 / 2561) GN=eno PE=3 SV=1 - [ENO_PORG3] | 19.01 | 16.24 | 2 | 5 | 5 | 45.8 |
| B2RHG3 | Minor fimbrium tip subunit Mfa3 OS=Porphyromonas gingivalis (strain ATCC 33277 / DSM 20709 / CIP 103683 / JCM 12257 / NCTC 11834 / 2561) GN=mfa3 PE=1 SV=1 - [MFA3_PORG3] | 18.10 | 20.40 | 1 | 5 | 5 | 50.0 |
| Q9S3R9 | Outer membrane protein 41 OS=Porphyromonas gingivalis (strain ATCC BAA-308 / W83) GN=PG_0695 PE=1 SV=2 - [OMP41_PORGI] | 13.12 | 14.58 | 1 | 5 | 5 | 43.4 |
| B2RHV8 | Phosphoenolpyruvate carboxykinase [ATP] OS=Porphyromonas gingivalis (strain ATCC 33277 / DSM 20709 / CIP 103683 / JCM 12257 / NCTC 11834 / 2561) GN=pckA PE=3 SV=1 - [PCKA_PORG3] | 12.82 | 10.65 | 2 | 4 | 4 | 59.4 |
| B2RJM1 | Hydroxylamine reductase OS=Porphyromonas gingivalis (strain ATCC 33277 / DSM 20709 / CIP 103683 / JCM 12257 / NCTC 11834 / 2561) GN=hcp PE=3 SV=1 - [HCP_PORG3] | 12.39 | 8.00 | 2 | 4 | 4 | 60.4 |
| B2RH58 | Major fimbrium tip subunit FimD OS=Porphyromonas gingivalis (strain ATCC 33277 / DSM 20709 / CIP 103683 / JCM 12257 / NCTC 11834 / 2561) GN=fimD PE=1 SV=1 - [FIMD_PORG3] | 10.40 | 5.97 | 1 | 4 | 4 | 75.8 |
| B2RGR2 | Serine hydroxymethyltransferase OS=Porphyromonas gingivalis (strain ATCC 33277 / DSM 20709 / CIP 103683 / JCM 12257 / NCTC 11834 / 2561) GN=glyA PE=3 SV=1 - [GLYA_PORG3] | 6.29 | 7.75 | 1 | 3 | 3 | 46.6 |
